# A strategy for synergistic ethanol yield and improved production predictability through blending feedstocks

**DOI:** 10.1186/s13068-020-01791-z

**Published:** 2020-09-05

**Authors:** Michael Persson, Mats Galbe, Ola Wallberg

**Affiliations:** Department of Chemical Engineering, Lund University, P.O. Box 124, 221 00 Lund, Sweden

**Keywords:** Ethanol, SSF, Substrate blending, Pretreated wheat straw, Saccharified wheat grain, Process integration, Hydrolysis, Fermentation, Blending synergy, Fermentation dynamics

## Abstract

**Background:**

The integration of first- and second-generation bioethanol processes has the potential to accelerate the establishment of second-generation bioethanol on the market. Cofermenting pretreated wheat straw with a glucose-rich process stream, such as wheat grain hydrolysate, in a simultaneous saccharification and fermentation process could address the technical issues faced during the biological conversion of lignocellulose to ethanol. For example, doing so can increase the final ethanol concentration in the broth and mitigate the effects of inhibitors formed during the pretreatment. Previous research has indicated that blends of first- and second-generation substrates during simultaneous saccharification and fermentation have synergistic effects on the final ethanol yield, an important parameter in the process economy. In this study, enzymatic hydrolysis and simultaneous saccharification and fermentation were examined using blends of pretreated wheat straw and saccharified wheat grain at various ratios. The aim of this study was to determine the underlying mechanisms of the synergy of blending with regard to the yield and volumetric productivity of ethanol.

**Results:**

Replacing 25% of the pretreated wheat straw with wheat grain hydrolysate during simultaneous saccharification and fermentation was sufficient to decrease the residence time needed to deplete soluble glucose from 96 to 24 h and shift the rate-limiting step from ethanol production to the rate of enzymatic hydrolysis. Further, a synergistic effect on ethanol yield was observed with blended substrates, coinciding with lower glycerol production. Also, blending substrates had no effect on the yield of enzymatic hydrolysis.

**Conclusions:**

The effects of substrate blending on the volumetric productivity of ethanol were attributed to changes in the relative rates of cell growth and cell death due to alterations in the concentrations of substrate and pretreatment-derived inhibitors. The synergistic effect of substrate blending on ethanol yield was attributed in part to the decreased production of cell mass and glycerol. Thus, it is preferable to perform simultaneous saccharification and fermentation with substrate blends rather than pure substrates with regard to yield, productivity, and the robustness of the process.

## Background

Bioethanol was estimated to constitute 63% of all biofuel production in 2018 and is expected to continue being an important element in the decarbonization of the transportation sector [1]. The bulk of the bioethanol that is on the market is produced from first-generation feedstocks, such as sugarcane and corn, whereas second-generation bioethanol, which is made from lignocellulosic feedstock, makes up a small share. However, a transition from first-generation to second-generation biofuels produced from agricultural, forestry, and municipal waste has been recommended, due to issues with direct and indirect land use change that have been linked to the production of first-generation biofuels [2]. Despite the interest in second-generation bioethanol, the transition from first-generation ethanol production to the second-generation process has been slow. Several issues related to production economy, such as conversion efficiency and capital expenditures, have been identified as some of the most significant barriers to the continued commercialization of second-generation bioethanol [3, 4].

A design choice that affects capital expenditures and the potential conversion efficiency of an ethanol plant is whether to operate it in a simultaneous saccharification and fermentation (SSF) or separate hydrolysis and fermentation (SHF) configuration. The SSF configuration requires shorter residence times versus SHF [5, 6]. In a techno-economic analysis of a softwood-based ethanol plant [7], the SSF configuration outperformed SHF economically. The improved process economics were partly attributed to lower capital expenditures that resulted from the shorter residence times required in SSF.

Ethanol yield is another important process parameter that must be considered to achieve acceptable process economics, because the cost of feedstock is one of the largest contributors to the price of biofuels [8], accounting for one-third of the total production costs [4]. Thus, maximizing the conversion efficiency of each step in the process is critical in achieving favorable process economics in second-generation ethanol production. The effects of the process configuration—SSF or SHF—on the process yield are unknown. Certain studies have reported higher ethanol yields for SSF [6, 7, 9], whereas others claim that SHF has better yields [5, 10]. Thus, a greater understanding of strategies for increasing ethanol yields in SSF processes is needed if the benefits of decreased residence times in SSF processes are to be capitalized. One method of improving the ethanol yield and productivity in an SSF process, which in techno-economic analysis has also been shown to have competitive process economics compared to traditional production methods [11], is the integration of first- and second-generation processes by substrate blending.

Certain cases of the integration of first- and second-generation SSF processes—specifically, the blending of starchy and lignocellulosic substrates—have been reported to outperform cases without substrate blending with regard to ethanol yield [12, 13]. These findings indicate the existence of a synergy that improves the ethanol yield, arising from blending these types of substrates in SSF. However, due to the high inter-sample variability that is often found in these types of processes and the small effects that have been reported, further examination is needed to verify this synergy and determine its underlying mechanism.

Presumably, the synergistic effect during SSF should originate from factors that affect enzymatic hydrolysis or the performance of the fermenting organism. Adding nonenzymatic protein during the enzymatic hydrolysis of second-generation materials improves its yields [14, 15]. Whole wheat grain, the first-generation material that is used in [12], has a high protein content (11.2% to 21.1%) [16], perhaps explaining the synergistic effect. Conversely, replacing some of the feedstock in a second-generation SSF process with a first-generation process stream changes several parameters that could alter the metabolism and growth of yeast, including the initial glucose concentration, as described by Monod kinetics [17]; the concentration of pretreatment-derived inhibitors [18, 19]; and the concentration of assimilable nitrogen sources, vitamins, and minerals [20]. Previous studies on blending first- and second-generation substrates did not determine whether the synergy was attributed to effects on enzymatic hydrolysis or yeast metabolism and growth [12, 13].

In this study, the effects of blending saccharified wheat grain (SWG) and pretreated wheat straw (PWS) on ethanol yield and productivity during SSF, using *Saccharomyces cerevisiae* as the fermenting organism, were examined. In addition, a separate set of hydrolysis experiments were performed according to design of experiment (DoE) methodology to determine the effect of substrate blending on enzymatic hydrolysis. Our aim was to determine the extent to which SWG can be used to mitigate the effects of pretreatment-derived inhibitors on ethanol productivity; verify the synergistic effect of blending SWG and PWS on ethanol yield; and attribute these effects to the fermenting organism or the enzymatic hydrolysis of the lignocellulosic material.

## Results

### Final ethanol yields in SSF

**Fig. 1:**
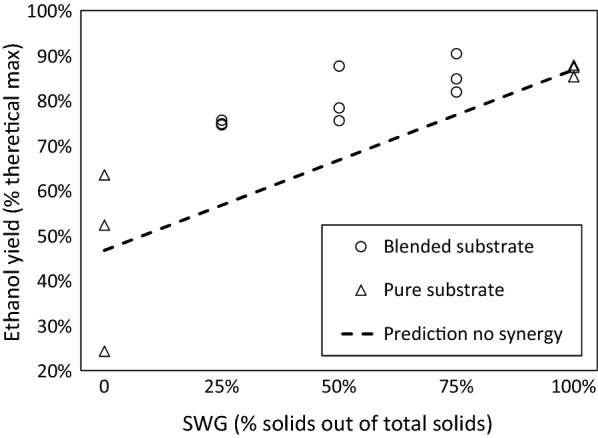
Measured and predicted ethanol yields after 96 h in SSF of SWG and PWS blends and pure substrate references

Figure 1 shows the SSF ethanol yields after 96 h for pure substrate reference cases and the cases with substrate blending. All blends resulted in ethanol yields by SSF that were higher than the values predicted in the absence of synergy. These data suggest that blending substrates has a synergistic effect on process yields. Further, all cases with SWG present had a smaller inter-sample spread in the yield after 96 h compared with pure PWS, suggesting that replacing some of the PWS with SWG stabilized the SSF.

The lowest yield was 24.3% after 96 h, occurring in a pure PWS sample, attributed to the incomplete consumption of glucose. In this case, after 96 h the residual concentration of glucose was 26.7 g/L. Because the concentration of ethanol continued to increase between 72 and 96 h, in this low-yield case, a higher ethanol yield would likely have been attained if the experiment had been allowed to progress. However, because a main argument for choosing the SSF process configuration was that it reduces the residence time that is required compared with the SHF configuration, longer operational times were beyond the scope of this study.

### Glucose consumption and rate limitations

**Fig. 2:**
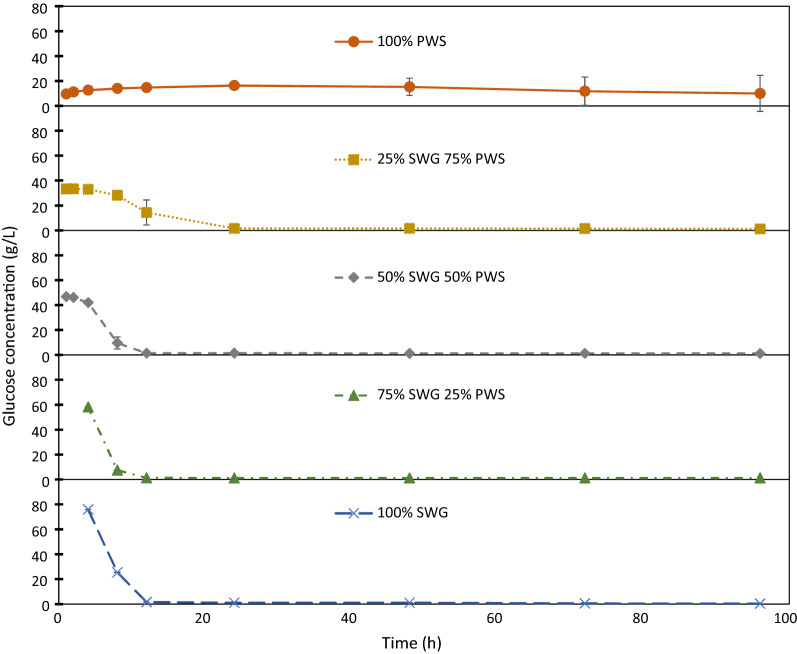
Concentration of soluble glucose in SSF broth over time. From the SSF of SWG and PWS blend experiments

**Fig. 3:**
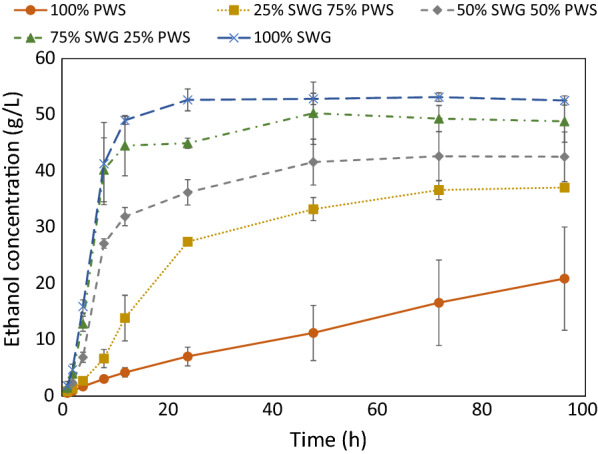
Concentration of ethanol in the SSF broth over time. From the SSF of SWG and PWS blend experiments

Figure 2 shows the changes in the concentration of glucose in the fermentation broth during the SSF of the SWG and PWS blends. The glucose consumption rate with pure PWS was initially slower than that at which glucan was hydrolyzed to glucose, as evidenced by the accumulation of glucose rather than its depletion. The concentration of soluble glucose dropped to below 2 g/L after 24 h for all cases in which SWG was present. In contrast, with pure PWS, it took 96 h for glucose concentrations to fall to comparable levels in 2 samples, whereas glucose accumulated for the entire duration in the remaining sample. Considering these data with the observation that ethanol concentrations continued to increase even after glucose concentrations declined to below 2 g/L when SWG was present, as shown in Fig. 3, we conclude that the rate-limiting conversion step shifted between these cases. Whereas all cases with SWG were rate-limited by the conversion of cellulose to glucose—i.e., by enzymatic hydrolysis—the pure PWS SSF was rate-limited by the conversion of glucose to ethanol—i.e., fermentation.

**Fig. 4:**
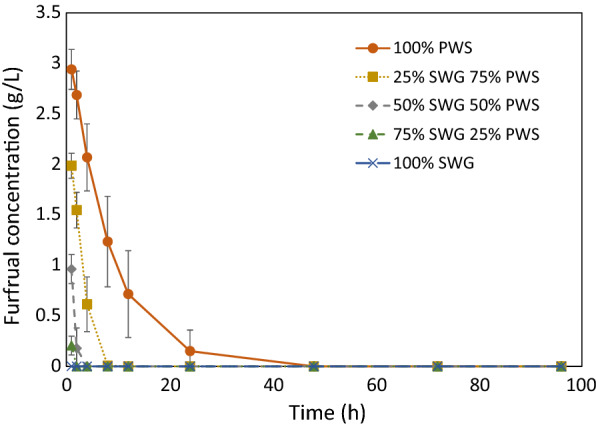
Concentration of furfural in the SSF broth over time. From the SSF of SWG and PWS blend experiments

Figure 4 shows the average concentration of furfural in the SSF broths during the SSF experiments. Whereas the concentration of furfural fell below the limit of detection in the first 12 h in all cases with SWG, it did so between 24 and 48 h with pure PWS. This finding indicates that a 25% reduction in the initial concentration of inhibitors—as seen comparing pure PWS (0:1) with the lowest proportion of SWG (1:3)—effects a 50% to 75% decrease in the time that is required for complete removal of furfural from the system.

### Glycerol production during SSF

**Fig. 5:**
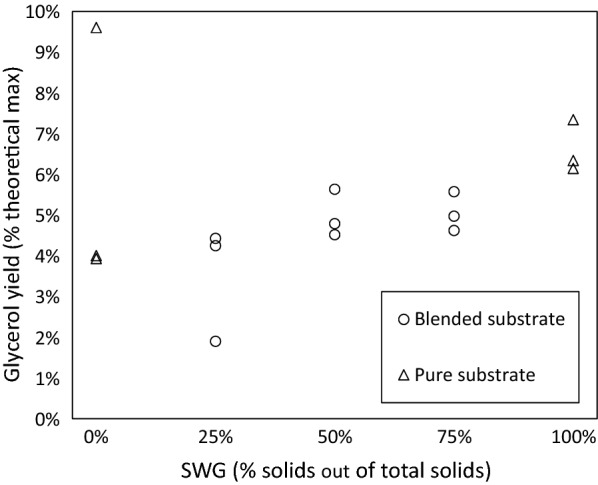
Glycerol yield in SSF with SWG and PWS blend experiments

To determine the underlying causes of the synergistic behavior with regard to ethanol yield (Fig. 1), the presence of the metabolic byproduct glycerol was measured. Figure 5 shows the final concentrations of glycerol in the SSF with the SWG and PWS blends. The lowest average concentration of glycerol was observed with an SWG:PWS ratio of 1:3. Further, excluding pure PWS, the average concentration of glycerol in the broth after 96 h increased as the fraction of SWG in the solids rose. Also, whereas pure PWS elicited a higher average glycerol yield than the 1:3 SWG:PWS ratio, it had the highest variance in glycerol yield of all cases, underscoring the importance of process stability when replacing some of the PWS with SWG. These results (Fig. 5) indicate that there is an optimal SWG:PWS ratio with regard to minimizing glycerol production. The dip in glycerol production could explain in part the synergistic behavior in ethanol yield.

**Fig. 6:**
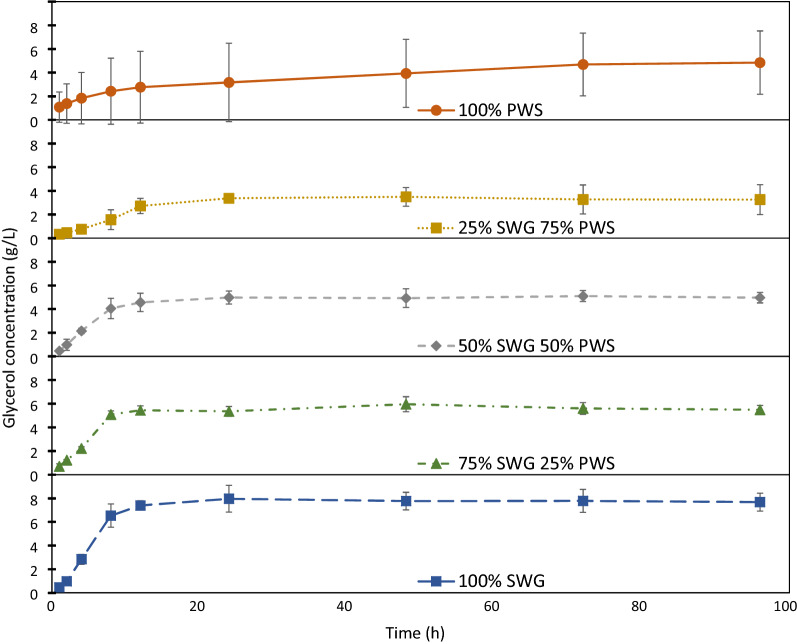
Concentration of glycerol in the SSF broth over time. From the SSF of SWG and PWS blend experiments

Figure 6 shows the concentration of glycerol in the fermentation broth during the SSF of the SWG and PWS blends. The increase in glycerol concentration plateaued after 24 h for all cases with SWG (1:3, 1:1, 3:1, 1:0) but continued steadily with pure PWS (0:1) throughout the experiment.

### Hydrolysis DoE

When fitting linear regression models to the hydrolysis yield data from the hydrolysis experiments, no model achieved a coefficient of determination that was higher than R^2^ = 0.117. The model with this value was the most complex, considering all of the main, quadratic, and interaction effects. None of the factors in this model had a significant effect (P < 0.1) on the hydrolysis yield. These results suggest that in the range of conditions in this study, there were no systematic changes in the hydrolysis yield of structural carbohydrates with regard to the mass loadings of SWG or PWS. In addition, there was no significant difference (p = 0.55) between the average hydrolysis yields under the DoE conditions (78.1 ± 8.0%)—i.e., the blended conditions—and the average yields with pure PWS (81.0 ± 0.4%).

## Discussion

The results of the SSF experiments, as seen in Fig. 1, indicate a synergistic effect on the final ethanol yield, due to the blending of SWG and PWS. In this case, synergy was defined as a positive deviation from the values that were predicted with the assumption that the final ethanol yields for blends would be a linear combination of those with pure substrate. This synergistic effect was supported by the finding that the final ethanol yield for all samples with blends exceeded the predicted values, consistent with previous studies that showed favorable yields when blending first- and second-generation substrates during SSF [12, 13].

In yeast, glycerol production is a means of reoxidizing NADH to NAD + to maintain the intracellular redox balance, a process in which glucose is consumed [21, 22]. As seen in Fig. 5, with the exception of pure PWS, higher-SWG blends resulted in greater average glycerol yields. Because glucose was the only substrate for ethanol production in the SSF experiments, any glucose that was diverted to the production of glycerol could be viewed as directly limiting the potential ethanol yield. There are several potential explanations for the changes in glycerol production with SWG (1:3, 1:1, 3:1, and 1:0).

One potential cause of glycerol production during anaerobic fermentation is the growth of yeast cells. The formation of yeast cells under anaerobic conditions creates a surplus of NADH [21, 23]. To maintain the intracellular redox balance of yeast during growth, the formation of 100 g of yeast is associated with the production 124.2 g glycerol [21]. The main factor that is expected to affect the final yield of yeast cell mass, when changing the SWG:PWS ratio, is the concentration of pretreatment-derived inhibitors that originate from the PWS, including furfural, hydroxymethylfurfural (HMF), and acetic acid. These substances inhibit cell growth [18, 19, 24], and furfural lowers the yields of yeast cell mass [24]. Thus, it is possible that the changes in final glycerol yield, with blended substrates and pure SWG, are attributed in part to changes in the amount of cell mass produced. Notably, in addition to the loss in glucose due to glycerol production, the higher concentrations of glycerol would thus be associated with further losses in glucose due to increased cell formation.

Another potential contributing factor to the changes in glycerol production is related to the mechanism by which inhibitors are mitigated in yeast. When furfural and HMF are present during fermentation using *S. cerevisiae*, cell growth and ethanol production are initially slowed while inhibitors are reduced to their corresponding alcohols by alcohol dehydrogenase [25]. This reduction is dependent on NADH, resulting in a surplus of NAD + , as shown for the reduction of furfural in *S. cerevisiae* [26]. The excess NAD + that is produced during this process reflects less of a need for glycerol production for the reoxidization of NADH, as posited by a study in which glycerol production fell in the presence of furfural [24]. This mechanism together with the argument that inhibitors decrease yeast production, and therefore glycerol production, both support the notion that the presence of inhibitory compounds in part explain the decline in glycerol production and increased ethanol yield.

In addition to the reoxidization of NADH, yeast can produce glycerol as an osmolyte to counterbalance extracellular osmotic pressure [27]. Based on the design of the SSF experiments, altering the SWG:PWS ratio entailed a change in the initial solute concentration. Considering the initial concentration of soluble sugars alone, the values in the SSF experiment ranged between 32 g/L (pure PWS) and 105.1 g/L (pure SWG). Thus, osmotic pressure might have been a factor in the production of glycerol. A previous study on the effects of extracellular osmotic pressure on the production of glycerol during anaerobic fermentation, using *S. cerevisiae* as the fermenting organism, found that varying the initial concentration of glucose between 50 and 200 g/L resulted in glycerol yields between 0.020 and 0.027 g/g [28]. However, this variation in yields did not appear to correlate with changes in glucose concentration. Because the initial solute concentrations in the SSF with the SWG and PWS blend were within the range in [28], the changes in glycerol production that we observed are unlikely to have resulted from a response to extracellular osmotic stress.

Another potential cause of the changes in final ethanol yield in the SSF experiments could be a change in the efficiency of the enzymatic hydrolysis. If shifting the SWG:PWS ratio affected the hydrolysis yield during SSF, the amount of available glucose would have been affected, in turn changing the attainable ethanol yield. Adding nonenzymatic protein during the enzymatic hydrolysis of lignocellulosic materials can increase hydrolysis yields [14, 15]. Because the crude protein content of the solids in the SWG was approximately 12%, we aimed to determine whether blending SWG and PWS would have an effect on the hydrolysis yield. However, as seen in the hydrolysis DoE experiments, no systematic changes in the hydrolysis yield with respect to changes in the mass loading of SWG or PWS were observed. A systematic response to a change in the SWG:PWS ratio would have been expected had blending SWG and PWS had a synergistic effect on the hydrolysis yield of structural carbohydrates.

The concentrations of protein in the experiments with the lowest amounts of SWG might have been sufficient to fully realize any beneficial effect on hydrolysis. However, as seen when comparing the overall average hydrolysis yields for all conditions in the hydrolysis DoE experiments, there were no significant differences from the yield with pure PWS. This finding suggests that the proteins in SWG do not behave like the proteins in previous experiments with regard to improving hydrolysis yields [14, 15]. Thus, we conclude that the experimental results show no evidence that SWG and PWS synergize in improving the enzymatic hydrolysis yield of structural carbohydrates in the range of conditions that we established.

### Rate limitations and process robustness

Two of the main observations in the SSF experiments relate to changes between pure PWS (0:1) and the presence of SWG (1:3, 1:1, 3:1, 1:0). With pure PWS, the conversion of glucose to ethanol was rate-limiting, whereas that of cellulose to glucose was rate-limiting in all other cases. In the cases with SWG, cellulose to glucose conversion rate limitation was evidenced by the rapid consumption of soluble glucose, followed by continued ethanol production for the duration of the experiment, indicating that glucose was released and converted to ethanol, even after measured concentrations of glucose were at the level of depletion. Conversely, with pure PWS, glucose to ethanol conversion rate limitation was reflected by the accumulation of soluble glucose, demonstrating the inability of the yeast to convert glucose at the rate that it was produced (Fig. 2).

The second observation relates to the robustness of SSF. With pure PWS (Fig. 1), the ethanol yield after 96 h had a higher spread compared with when SWG was present. The change in robustness was further exemplified by the variation in the capacity of the yeast to assimilate soluble glucose during the experiment with pure PWS (Fig. 2).

A potential explanation for both observations relates to the dynamics of yeast viability during the experiments. Changing the SWG:PWS ratio is expected to affect the yeast mass yield during SSF. However, this ratio should have an even greater effect on the general dynamics of yeast viability. When the SWG:PWS ratio shifts, the initial concentration of soluble glucose and the concentration of pretreatment-derived inhibitors are altered. The case with pure SWG had the highest initial concentration of soluble glucose and the lowest concentration of pretreatment-derived inhibitors, whereas pure PWS resulted in the opposite pattern. With blended substrates, the concentration of glucose and inhibitors changed linearly between these endpoints.

The specific growth rate of microorganisms is linked to the substrate concentration, according to Monod kinetics [17], with greater substrate concentrations resulting in higher growth rates. In contrast, pretreatment-derived inhibitors lower specific growth rates concentration-dependently [18, 24]. Also, growth can be halted completely when several inhibitors are added simultaneously [29]. Further, at sufficient concentrations, pretreatment-derived inhibitors may kill yeast cells [18].

Considering these findings, the observations in the SSF experiments could be explained as follows. With pure PWS, high concentrations of inhibitors and the initial unavailability of substrate could have resulted in low cell growth rates relative to that of cell death. A high rate of cell death versus growth would have resulted in a net loss of viable cells in the presence of pretreatment-derived inhibitors. Because the rate at which the inhibitors can be decreased by yeast depends on the total concentration of yeast [18], losing viable yeast cells would result in slower conversion of inhibitors.

This model is consistent with the results in Fig. 4, in which the time for complete elimination of furfural rose 2- to 4-fold with pure PWS compared with the second highest initial concentration of inhibitors (1:3). That volumetric ethanol productivity depends on the concentration of viable yeast and is negatively affected by pretreatment-derived inhibitors [18, 24, 30] explains why fermentation was the rate-limiting conversion step with pure PWS. Thus, the shift in the SWG:PWS ratio from 0:1 to 1:3 likely changed the cell growth and death rates sufficiently to switch the rate -limiting conversion step from fermentation to hydrolysis.

Further, had the growth and death rates been similar, it could explain the instability with pure PWS, because any small change that affects either rate would be sufficient to alter the overall trajectory of viable cell mass in the system. A change of this nature could have an escalating effect on the system in either direction—higher growth would increase the cell mass, in turn mitigating the inhibitors more easily, whereas greater cell death would have the opposite effect.

These findings have implications for improving the robustness and reliability of SSF. If the outcome of SSF cannot be reliably predicted, ethanol producers run the risk of economic losses due to poor conversion of the feedstock and increased lead times. The findings in this study suggest that replacing part of the feedstock in a PWS-based SSF process with SWG is a viable strategy for stabilizing the performance of the yeast when feedstock with high concentrations of inhibitors is used. There are many potential benefits of integrating an ethanol plant using wheat straw as feedstock with an ethanol plant that uses wheat grain as its feedstock. Doing so obviates the need for detoxification of PWS upstream of the fermentation step; reduces capital expenditures related to fermenters, because volumetric productivities are less affected by the dynamics of cell growth; and lower process costs that are associated with yeast production, because less cell mass would be required to achieve similar productivities.

## Conclusions

An ethanol production plant that is configured to blend SWG and PWS in a combined SSF process addresses many of the issues that arise in the biochemical conversion of wheat straw to ethanol. Our main finding was that replacing a small portion of the solids in a pure PWS SSF with SWG mitigates the negative effect of pretreatment-derived inhibitors, on the volumetric productivity of ethanol. Further, blending SWG and PWS showed slight synergistic behavior with regard to ethanol yield, most likely due to changes in the production of glycerol and cell mass—not to systematic changes in the enzymatic hydrolysis of structural carbohydrates.

## Materials and methods

### Wheat straw and wheat grain mixture

Winter wheat straw was collected near Køge, Denmark. The straw was air-dried to a moisture content below 10% before it was chopped into 5–10 cm pieces and stored at room temperature before pretreatment. A grain meal mixture that comprised 90% wheat, 5% triticale, and 5% barley was kindly provided by Lantmännen Agroetanol.

### Saccharified wheat grain

The grain mixture was saccharified in a 2-step enzymatic hydrolysis procedure, consisting of a liquefaction and saccharification step. A 20-L evaporator (Rotavapor^®^ R-153; Büchi Labortechnik AG, Flawil, Switzerland) was used for both steps of the hydrolysis. The grain mixture was mixed with water to a dry matter content of approximately 25%. In the first step, the grain slurry was supplemented with 0.5 ml/kg dry matter of *Bacillus licheniformis* α-amylase (Sigma Aldrich, Denmark) and liquefied at 90 °C and pH 5.5 for 3 h. In the second step, the slurry was saccharified with 1 ml/kg dry matter of *Aspergillus niger* amyloglucosidase (Sigma Aldrich, USA), which had an activity of 260 U/ml, at 60 °C and pH 5 for 24 h. After saccharification, the SWG was frozen and stored before use.

### Pretreated wheat straw

The wheat straw was soaked overnight in an aqueous solution of 0.2% H_2_SO_4_ at a solid:liquid ratio of 1:20 in sealed buckets. Then, the straw was pressed in a 25-L filter press (Tinkturenpressen HP25M, Fischer Maschinenfabrik GmbH, Germany) to a dry matter content of 44%.

Next, the wheat straw was steam-pretreated in a 10-L reactor (Process & Industriteknik AB, Kristianstad, Sweden), as described in [31]. The conditions for the steam pretreatment were 190 °C for 10 min using saturated steam, based on the optimized conditions for the steam pretreatment of wheat straw [32]. The pretreated wheat straw was stored in sealed buckets at 4 °C before use.

### Analytical methods

#### Compositional analysis

The carbohydrate and lignin content of the wheat straw and PWS was determined per National Renewable Energy Laboratory (NREL) standards [33]. The monomeric and oligomeric sugar content and the content of the degradation products in the liquid fraction of the PWS were measured per standard NREL procedures [34].

#### Crude protein content

The crude protein content in the wheat grain and SWG was estimated using a nitrogen correction factor of 6.25, multiplied by the total nitrogen content. Total nitrogen was measured on an N/Protein Analyzer (Flash EA 1112 Series, Thermo Electron S.p.A., Rodano, Italy), equipped with a carbon trap (soda lime), water trap (silica gel), CuO and Pt/Al2O3 catalysts, and a Teflon and activated carbon separation column, using a thermal conductivity detector. Prior to analysis, the samples were dried in a 105 °C convection oven. Before separation, the samples were combusted in the presence of oxygen by Dumas method to reduce the nitrogen in the samples to nitrogen gas [35]. Aspartic acid was used as a calibration standard.

#### Composition of material

The results of the compositional analysis of the PWS are listed in Table 1. The glucan content in the PWS was 68.3 ± 0.6%, consistent with previous findings on the pretreatment of wheat straw under the same conditions [12, 36]. The furfural (3.8 g/L) and HMF content (0.5 g/L) in the PWS liquid was also in the range of reported values under similar pretreatment conditions [36, 37]. The SWG was analyzed for its glucose content and concentration of insoluble residues, but the composition of the latter was not examined, because we deemed that any carbohydrates in the residues would have had a minor impact on the final soluble glucose concentrations. This assumption was based on the finding that starch-free residues of wheat meal have a glucan content of 17.5 ± 0.1% [12]. Further, another study reported that the glucose yields from the enzymatic hydrolysis of the structural glucan in untreated starch-free residues from wheat meal were 2-4 times lower than what could be achieved by pretreating the starch-free residues [38]. These data indicate that any release of glucan from the starch-free residue would have a negligible impact on the measurements during SSF compared with the contribution from glucan from structural carbohydrates in the PWS and soluble glucose in the SWG liquid.

### Analysis of SSF and hydrolysis samples

The liquid samples from the SSF and hydrolysis experiments were first centrifuged in Eppendorf tubes at 13,000 rpm for 10 min. The supernatant was then passed through 0.2-μm syringe filters and stored in a freezer before analysis. The ethanol, glycerol, organic acid, and carbohydrate content of the liquid samples from the SSF experiments was determined by high-performance liquid chromatography (HPLC) using a refractive index detector (RID-10A, Shimadzu, Kyoto, Japan), with an Aminex HPX-87H column for separation. The carbohydrate content of the hydrolysis samples was determined by HPLC using a refractive index detector (RID-10A, Shimadzu, Kyoto, Japan), with an Aminex HPX-87P column for separation.

### SSF of SWG and PWS blends

The SSF experiments were performed in 2-L Labfors bioreactors (Labfors 3,Infors AG, Bottmingen Switzerland) with a working mass of 900 g. The fermentors and equipment were sterilized prior to the addition of PWS and SWG. The temperature during SSF was maintained at 35 °C during the experiment. The pH was set to 5 and controlled automatically by the addition of a 10% w/w ${\text{NaOH}}$ solution. The mass loadings of PWS and SWG were set to achieve the desired PWS solids:SWG solids ratio. Deionized water was added to dilute the substrate blend to a total solids loading of 15% for all cases. A nutrient solution was prepared separately and added to the fermentors before inoculation to a nutrient concentration of 0.5 g/L $\left( {{\text{NH}}_{ 4} } \right)_{2} {\text{HPO}}_{4}$, 0.025 g/L ${\text{MgSO}}_{4} \cdot 7{\text{H}}_{2} {\text{O}}$, and 1 g/L yeast extract. Ethanol Red yeast (Lesaffre, France) was used at an initial concentration of 3 g/L of dry yeast. Before inoculation, the dry yeast was activated with deionized water, at 5 times the solids weight of the yeast, and incubated at 35 °C for approximately 30 min. A Cellic Ctec2 enzyme preparation was used at 15 FPU/g glucan. The glucan content during fermentation was based on an estimate of the combined contributions of glucan from the PWS and SWG.

### Experimental design: SSF

To determine whether blending SWG with PWS during SSF would induce a synergistic response with regard to ethanol yield and productivity, 5 experimental conditions were applied: 3 blended cases and 2 for a pure substrate reference. The ratios of SWG to PWS in the 3 blends were 1:3, 1:1, and 3:1, on a dry weight basis. In the first pure references case, only SWG was added (1:0), and the second reference consisted only of PWS (0:1). The total initial solids loading was kept constant at 15% for all conditions. The SSF experiments were performed in triplicate.

### Synergy model

The final ethanol yields for the blended substrate cases in the SSF experiments were compared with a prediction of the final ethanol yield. The prediction was based on the assumption that the final ethanol yield in the blended cases would be a linear combination of the ethanol yield in the pure substrate reference cases. The predicted values for the ethanol yield were calculated per Eq. 1:

1 $$Y_{{{\text{EtOH,}}\,{\text{pred}}}} = Y_{{{\text{PWS,}}\,{\text{pure}}}} \cdot X_{\text{PWS}} + Y_{{{\text{SWG, }}\,{\text{pure}}}} \cdot X_{\text{SWG}} ,$$

where $Y_{{{\text{EtOH,}}\;{\text{pred }}}}$ is the predicted final ethanol yield, $Y_{{{\text{PWS,}}\,{\text{pure}}}}$ is the average final ethanol yield for pure PWS, $X_{\text{PWS}}$ is the fraction of PWS in the total solids, $Y_{{{\text{SWG,}}\,{\text{pure}}}}$ is the average final ethanol yield for the pure SWG, and $X_{\text{SWG}}$ is the fraction of SWG in the total solids. If the yields in the blended substrate cases were higher or lower than the predicted yields, substrate blending would be considered to have a synergistic or antagonistic effect on the final ethanol yield, respectively.

### Enzymatic hydrolysis DoE

To separate the effects of substrate blending on enzymatic hydrolysis from those on fermentation, a separate set of hydrolysis experiments were performed in 50-ml Falcon tubes with a working mass of 20 g per sample. The experiment was designed as a 3-level, 2-factor full factorial experiment with 4 centerpoint replicates. The 2 factors were the solids loadings of SWG and PWS, respectively. The experimental design can be seen in Table 2. A reference hydrolysis experiment with pure PWS was also performed in triplicate, using the same procedure as in the other hydrolysis experiments. The total solids load in the pure PWS reference case was set to 10%.

**Table 1:** Composition of PWS and SWG

PWS					SWG	
WIS		Liquid			Liquid	
Components	Percentage of DM	Components	Oligosaccharides	Monosaccharides	Components	
Glucan	68.3 ± 0.6	*Sugars, g/L*			*Soluble solids, g/L*	
Mannan	0.5 ± 0.4	Glucose	BDL*	4.8	Glucose	205.4 ± 3.3
Xylan	4.3 ± 0.01	Mannose	BDL*	1.0	*Insoluble solids, g/L*	
Galactan	0.3 ± 0.3	Xylose	59.8	28.2	Crude protein	32.5 ± 0.7
Arabinan	0.6 ± 0.2	Galactose	BDL*	3.5	Residual mass	22.3 ± 1.4
ASL	0.9 ± 0.0	Arabinose	BDL*	4.5		
AIL	28.7 ± 0.5	*Inhibitors, g/L*				
Total ash	5.1 ± 0.0	Acetic Acid	4.6			
		Furfural	3.8			
		HMF	0.5			

Composition of water-insoluble solids (WIS) in PWS is expressed as percentage of WIS dry matter. Components dissolved in PWS liquid are expressed as concentrations. All components of SWG are expressed as concentrations.* below detection limit

**Table 2:** Experimental design for enzymatic hydrolysis DoE experiment

Condition	PWS solids mass loading (% of total mass), %	SWG solids mass loading (% of total mass), %
1	2.5	2.5
2	5.0	2.5
3	7.5	2.5
4	2.5	5.0
5	5.0	5.0
6	7.5	5.0
7	2.5	7.5
8	5.0	7.5
9	7.5	7.5
10	5.0	5.0
11	5.0	5.0
12	5.0	5.0

The experiment was initiated with the addition of a Cellic Ctec2 (Novozymes, Denmark) enzyme preparation. The hydrolysis was carried out in a hybridization incubator (Combi-H12, FINEPCR, Seoul, Korea) for 96 h at 35 °C with 7-mm-diameter steel ball bearings for mixing. Fifteen FPU/g glucan of enzymes were added during hydrolysis, the glucan content of a sample was estimated as the combination of PWS and SWG glucan. The temperature (35 °C) was chosen to replicate the conditions used in the SSF experiment. Penicillin and streptomycin were added at 10,000 units/L and 10 mg/L, respectively. To maintain a stable pH during the hydrolysis, 50 mM citrate buffer, pH 4.8 was added when the samples were diluted to the desired solids loading according to the experimental design.

### Response surface regression

The data from the hydrolysis experiment were fitted to a response surface model by linear regression. In addition to the 12 sample points in the full factorial experiment, supplementary replicates of certain points were obtained, amounting to a total of 33 data points in the range of the full factorial design. The resulting dataset was used for the linear regression and can be seen in Additional file 1. The response variable in the regression analysis was the hydrolysis yield of structural carbohydrates, as defined in Eq. 2:

2 $$Y_{\text{hyd}} = \frac{{m_{{{\text{Glu,}}\,{\text{fin}}}} - m_{{{\text{Glu,}}\,{\text{init}}}} }}{{m_{{{\text{Glucan,}}\,{\text{tot}}}} \cdot X_{\text{Anhydro}} }},$$

where $Y_{\text{hyd}}$ is the final glucan hydrolysis yield; $m_{{{\text{Glu,}}\,{\text{fin}}}}$ is the mass of soluble glucose at the end of the experiment; $m_{{{\text{Glu,}}\,{\text{init}}}}$ is the mass of soluble glucose at the start of the experiment; $m_{{{\text{Glucan,}}\;{\text{tot}}}}$ is the mass of glucan in the sample at the start of the experiment, based on the mass of SWG and PWS that were added to the sample; and $X_{\text{anhydro}}$ is an anhydro correction of 1.11 for the hydrolysis of glucan to glucose. Main effects, interaction effects, and quadratic effects were considered during the fitting of the model.

## Supplementary information

**Additional file 1.** Conditions and hydrolysis yields of structural carbohydrates for enzymatic hydrolysis experiments within boundaries of DoE conditions and with pure PWS reference

## Data Availability

All data generated or analyzed during this study are included in this published article and its supplementary information files.

## Abbreviations

SSF: Simultaneous saccharification and fermentation
SHF: Separate hydrolysis and fermentation
SWG: Saccharified wheat grain
PWS: Pretreated wheat straw
DoE: Design of experiment
HMF: Hydroxymethylfurfural
HPLC: High performance liquid chromatography
