# Supplementary material for: A strategy for synergistic ethanol yield and improved production predictability through blending feedstocks
Source: Biotechnol Biofuels. 2020 Sep 5;13:156. doi: 10.1186/s13068-020-01791-z (PMC7487856; doi:10.1186/s13068-020-01791-z)
Supplement: Supplementary file 1 — Additional file 1. Conditions and hydrolysis yields of structural carbohydrates for enzymatic hydrolysis experiments within boundaries of DoE conditions and with pure PWS reference [file 13068_2020_1791_MOESM1_ESM.docx]

# Additional material S1

S1 A

Conditions and hydrolysis yields of structural carbohydrates for enzymatic hydrolysis experiments within boundaries of DoE conditions

| Sample | PWS solids  (% of total mass) | SWG solids  (% of total mass) | Hydrolysis yield |
| --- | --- | --- | --- |
| 1 | 2.5% | 2.5% | 80% |
| 2 | 2.5% | 5.0% | 91% |
| 3 | 2.5% | 7.5% | 79% |
| 4 | 5.0% | 2.5% | 86% |
| 5 | 5.0% | 5.0% | 83% |
| 6 | 5.0% | 7.5% | 69% |
| 7 | 7.5% | 2.5% | 84% |
| 8 | 7.5% | 5.1% | 82% |
| 9 | 7.5% | 7.5% | 85% |
| 10 | 5.0% | 5.0% | 88% |
| 11 | 5.0% | 5.0% | 80% |
| 12 | 5.0% | 5.0% | 82% |
| 13 | 2.5% | 5.0% | 90% |
| 14 | 5.0% | 2.5% | 84% |
| 15 | 5.0% | 5.0% | 80% |
| 16 | 7.5% | 7.5% | 82% |
| 17 | 5.0% | 5.0% | 84% |
| 18 | 5.0% | 5.0% | 85% |
| 19 | 2.5% | 2.5% | 78% |
| 20 | 2.6% | 5.0% | 69% |
| 21 | 2.5% | 7.5% | 65% |
| 22 | 5.0% | 2.5% | 72% |
| 23 | 5.0% | 5.0% | 70% |
| 24 | 5.0% | 7.5% | 65% |
| 25 | 7.5% | 2.5% | 68% |
| 26 | 7.5% | 5.0% | 63% |
| 27 | 5.8% | 5.8% | 67% |
| 28 | 5.0% | 5.3% | 71% |
| 29 | 5.0% | 5.0% | 67% |
| 30 | 7.5% | 7.5% | 83% |
| 31 | 5.0% | 5.0% | 85% |
| 32 | 5.0% | 5.0% | 83% |
| 33 | 5.0% | 5.0% | 84% |

S1 B

Conditions and hydrolysis yields of structural carbohydrates for enzymatic hydrolysis experiments with pure PWS reference case

| Sample | PWS solids  (% of total mass) | SWG solids  (% of total mass) | Hydrolysis yield |
| --- | --- | --- | --- |
| 1 | 10% | 0% | 81% |
| 2 | 10% | 0% | 81% |
| 3 | 10% | 0% | 81% |
